# Residual Chlorine Interaction with Microelements in Plants Applied for Phytoremediation in Rain Gardens

**DOI:** 10.3390/plants14131957

**Published:** 2025-06-26

**Authors:** Ieva Andriulaityte, Marina Valentukeviciene, Viktoras Chadysas, Antonina Kalinichenko

**Affiliations:** 1 Department of Environmental Protection and Water Engineering, Faculty of Environment Engineering, Vilnius Gediminas Technical University, LT-10223 Vilnius, Lithuania; ieva.andriulaityte@vilniustech.lt; 2 Department of Mathematical Statistics, Faculty of Fundamentals Science, Vilnius Gediminas Technical University, LT-10223 Vilnius, Lithuania; viktoras.chadysas@vilniustech.lt; 3 Institute of Environmental Engineering and Biotechnology, University of Opole, 45-040 Opole, Poland; akalinichenko@uni.opole.pl

**Keywords:** stormwater, plants, phytoremediation, residual chlorine, microelements

## Abstract

Stormwater pollution from residual chlorine after outdoor disinfection with sodium hypochlorite is an increasing environmental challenge due to its potential negative impact on aquatic ecosystems. Even at low concentrations, residual chlorine can disrupt the stability of water ecosystems. In this regard, stormwater treatment requires innovative and green solutions such as green infrastructure (rain gardens) using the plant phytoremediation technique to reduce the amount of residual chlorine. This study explores the interactions between residual chlorine retained by plants in a rain garden and different microelements. Selected plants were analyzed via spectroscopy, and possible interactions with elements such as chlorine (Cl), phosphorus (P), zinc (Zn), iron (Fe), calcium (Ca), potassium (K), nickel (Ni), silicon (Si), manganese (Mn), magnesium (Mg), chromium (Cr), and cadmium (Cd) were determined using Python-based analysis. Chlorine presented significant positive correlations with cadmium (0.39–0.53) and potassium (0.51–0.55), while negative correlations were found between silicon and chlorine (−0.48–−0.54) and chlorine and iron (−0.45–−0.51). The correlations between chlorine and microelements suggest both common uptake mechanisms and mutual interactions. These results provide a better understanding of the behavior of chlorine in rain gardens and its interactions with other materials, which is especially valuable for designing green infrastructure. This research can help to develop sustainable solutions that reduce environmental pollution and strengthen urban adaptation to climate change.

## 1. Introduction

Stormwater created from rain and snow precipitation that has not infiltrated the ground is one of the primary sources of environmental pollution [1,2]. Most stormwater runoff is generated in urbanized areas, which are covered by impermeable surfaces (such as asphalt, concrete, etc.), and contains various pollutants that can be directly transported into water bodies. This results in toxic effects in water ecosystems and can significantly disrupt the ecological balance of aquatic flora and fauna [3,4,5]. In this regard, sustainable stormwater management has become an important environmental and urban issue. Studies have indicated that improper stormwater management causes extreme environmental challenges, such as heavy rains, floods, long-term drought, erosion, infrastructure deterioration, and water body contamination [6,7,8]. According to the European Environment Agency, extreme climate conditions caused economic losses of EUR 738 billion in the European Union between 1980 and 2023, with more than EUR 162 billion (22%) in 2021–2023 [9]. As an example, DANA phenomena [10] have become a serious warning sign of the need to strengthen urban infrastructure and improve preparedness for extreme weather events, especially in light of the impacts of climate change. Traditional stormwater treatment thus requires an integrated and inclusive approach to ensure the green transition, to meet sustainable development goals, principles of circular economy and European Green Deal, as well to solve the issue of water scarcity [11,12,13]. Stormwater treatment integrating green infrastructure into existing gray infrastructure is highly recommended by the European Commission [14]. Studies have shown that green and blue technologies have significant potential to reduce water pollution and protect rivers and lakes [15,16,17,18,19].

The current research is focused on investigating the release of residual chlorine into stormwater after the disinfection of outdoor surfaces. Research has shown that outdoor surfaces are most commonly disinfected with substances containing chlorine or chlorine compounds, such as sodium hypochlorite [20]. Sodium hypochlorite is commonly used for cleaning outdoor surfaces in places that must be constantly disinfected to prevent the spread of infections and viruses, such as spas, nursing homes, outdoor restaurants, and other recreational spaces [21,22,23]. Outdoor disinfection using sodium hypochlorite solutions leads to residual chlorine in the environment [24]. Sodium hypochlorite is a strong oxidizer with clearly expressed corrosive properties and high toxicity in water ecosystems [25,26]. The World Health Organization recommends a concentration of 1000 ppm sodium hypochlorite for surface disinfection [27]. Some countries used doses of sodium hypochlorite that were several times greater for surface disinfection in an attempt to stop the spread of the pandemic [28,29]. Residual chlorine, even at low concentrations, has a toxic impact on aquatic flora and fauna, adversely affecting their growth, reproductive capacities, and survival rate [30]. Research found that the permanent introduction of pollutants at low concentrations increases their presence in water bodies and might harm the biological components of water ecosystems [31]. This finding suggests the need to investigate environmentally friendly and new stormwater treatment methods to retain residual chlorine. Stormwater management using low-impact development systems, such as rain gardens, permeable pavements, and green roofs, has been applied to reduce stormwater contamination and solve water scarcity issues. Chlorinated swimming pool water can be reused through green infrastructure for irrigation and other needs [32]. This indicates that the residual chlorine retention due to plant phytoremediation might be an innovative solution to reduce stormwater contamination by disinfectants containing chlorine compounds.

Phytoremediation is based on the idea of removing pollutants from stormwater using the capacities of plants to absorb, accumulate, and decompose harmful contaminants [33,34]. This treatment technology is recommended by the European Commission as an effective strategy to reduce stormwater contamination. Studies have shown that phytoremediation using various plants and tree–based phytoremediation techniques effectively reduces environmental pollution [35,36]. Different phytoremediation technologies can be applied for stormwater treatment, including green areas such as wetlands, biofilters, and sustainable urban solutions such as green roofs or rain gardens [37,38,39,40]. Research has highlighted that the efficiency of phytoremediation measures depends on the selection of appropriate plant species [41]. In this regard, it is essential to evaluate the key plants’ capacities, such as their pollutant tolerance, accumulation capacities, growth rate, location conditions, and suitable plant species. Plants retain stormwater pollutants, decomposing or accumulating them in their roots, stems, and leaves. Studies have shown that fast-growing plants produce a larger amount of biomass, thereby ensuring the more efficient removal of contaminants. Research has suggested using native plants for stormwater treatment via phytoremediation. These plants are adapted to local conditions, do not require special care, and have better adaptation, better ecosystem stability, a lower risk of invasion, and improved efficiency [42,43,44,45].

Green infrastructure, such as rain gardens, reduces pollution by retaining and filtering stormwater. This research is a continuation of an analysis focused on investigating the interactions between chlorine and microelements in plants, reflecting their ability to reduce the release of chlorine into the environment. Previous studies have shown that various filter and drainage materials can retain chlorine [24,46]. Studies have also revealed that chlorine interacts with various elements, such as potassium (K), iron (Fe), copper (Cu), and zinc (Zn), which can affect the degradation of surface cover. This study aims to investigate the interactions between chlorine and microelements in plants, in order to assess plants’ ability to retain chlorine before stormwater enters the environment. The research results are expected to reveal which plants absorb chlorine most effectively and how the interaction of microelements with chlorine ions contributes to their retention mechanism. This will allow for the creation of more accurate recommendations for the selection of plant species for rain gardens in order to effectively reduce chlorine pollution. Rain gardens can be an effective tool for stormwater treatment and secondary water use.

The main aim of the present research is to assess the possibility of integrating green infrastructure (rain garden) and the principles of phytoremediation to treat stormwater polluted by residual chlorine. To achieve this goal, the study sets following objectives: to investigate the interaction of residual chlorine with microelements in plants after surface disinfection, to assess the ability of plants to retain chlorine and their potential to reduce chlorine pollution in stormwater, to determine the relationships between residual chlorine and certain microelements, to evaluate their correlation impact on plants, and to provide recommendations on the selection of plant species for rain gardens for effective stormwater treatment. This study’s novelty is that residual chlorine’s interactions with certain plant microelements were evaluated for the first time after stormwater contamination with disinfectants. The present research combines the principles of phytoremediation with the methods of microelement analysis to achieve sustainable and green stormwater treatment solutions. This study uncovers the antagonistic effects of chlorine and some microelements, which may be significant for plant selection in the design of green infrastructures. This study contributes to solving the problem of water scarcity by proposing sustainable measures to reduce chlorine pollution.

## 2. Results and Discussion

### 2.1. Correlation Analysis

**Figure 1:**
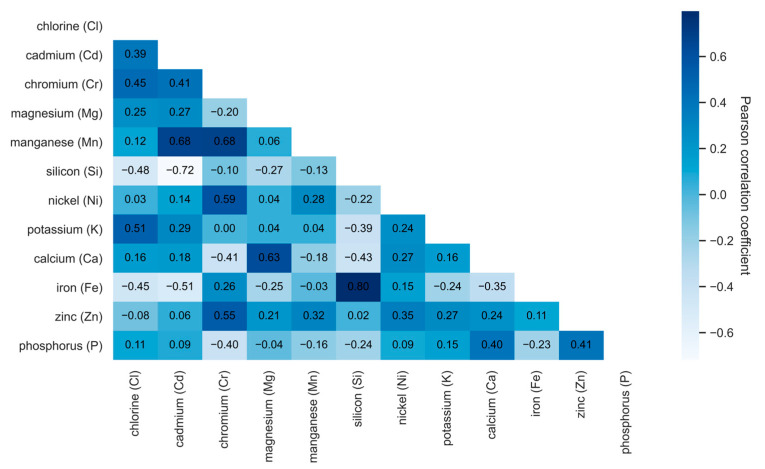
Heatmap of Pearson correlation coefficients based on the original dataset showing the interaction between chlorine (Cl) and selected microelements in plant samples. Stronger positive correlations are shown in dark blue, while weak or negative correlations are indicated by light or white tones.

**Figure 2:**
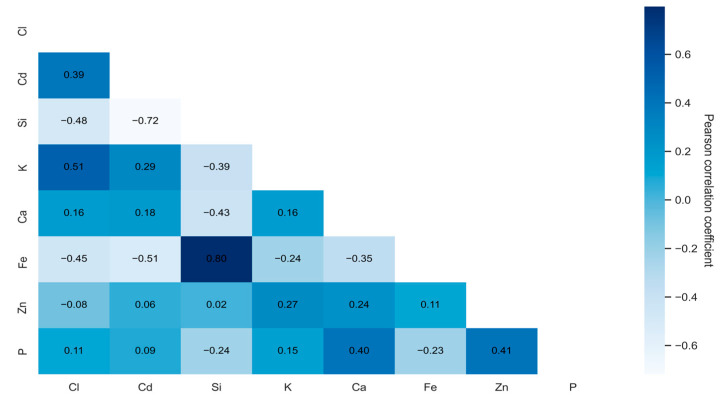
Heatmap of correlations found between chlorine and other microelements using a filtered dataset (with missing values removed). This visualization highlights significant positive (Cl-K and Cl-Cd) and negative (Cl-Si and Cl-Fe) relationships.

**Figure 3:**
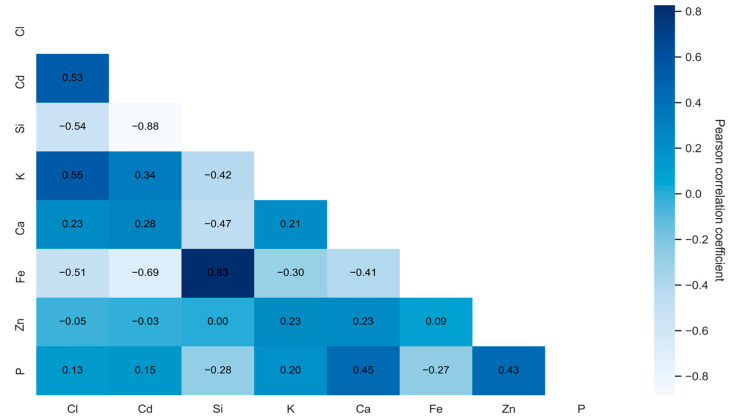
Correlation matrix of aggregated data based on average sample values. The matrix confirms previous trends and visualizes statistically significant interactions between chlorine and other elements.

The correlational analysis was conducted using the following steps: first, data analysis was performed on the original set, ignoring blank values. Second, to preserve as much meaningful information as possible, missing values were estimated and removed to avoid negative impacts on the results’ accuracy. Third, an analysis was performed on summary data using mean values across the sample. This approach ensured that each observation represented an average measurement, rather than individual samples. The obtained data revealed the interactions between chlorine (Cl) and certain microelements in plants, including phosphorus (P), zinc (Zn), iron (Fe), calcium (Ca), potassium (K), nickel (Ni), silicon (Si), manganese (Mn), magnesium (Mg), chromium (Cr), and cadmium (Cd), as shown in Figure 1, Figure 2 and Figure 3.

Figure 1 shows the interactions between chlorine (Cl) and different microelements, with the correlation coefficients ranging from −0.6 to 0.6. The strength and direction of these interactions are visually expressed on a color scale, where dark blue represents stronger positive correlations, lighter colors indicate weak correlations, and white represents negative correlations. Positive correlations were obtained between chlorine (Cl) and cadmium ((Cd): 0.39) and chromium ((Cr): 0.45). This indicates that, when increasing the chlorine in plants, the amounts of cadmium and chromium also increase. The scientific literature has indicated that cadmium reduces plants’ uptake of Fe and Zn and might impact the transport and uptake of Ca, P, Mg, K, and Mn [47,48,49]. On the contrary, in the case of chlorine, it increases with increasing Cd content. Cadmium is naturally found in phosphate minerals, which are used to make phosphorus fertilizers. When such fertilizers are used, cadmium accumulates in the soil, contributing to increased Cd concentrations in plants. This indicates that elements with a strong positive correlation with chlorine (Cl) might have a common origin or similar uptake and accumulation mechanisms in plant tissues. A strong positive correlation (0.68) was observed between manganese (Mn), cadmium (Cd) and cromium (Cr), as well (0.80) silicon (Si) and iron (Fe), which may impact the interaction of these elements in plants’ physiological processes. Phosphorus (P) and zinc (Zn) also showed a significant positive correlation of 0.41. Thus, correlations between certain microelements might be determined by several factors, such as environmental conditions or specific plant physiological responses to environmental stress or nutrient imbalances, as well as phytoremediation features inherent to the investigated plant species, which determine the selective accumulation of elements or mechanisms of their interaction [50,51].

Meanwhile, a negative correlation indicates an inverse interaction between chlorine and specific elements, which may be related to different uptake or transport mechanisms of these elements in plants. Figure 1 shows that chlorine (Cl) was correlated negatively with iron (Fe) and silicon (Si), with coefficients of −0.45 and −0.48, respectively. This might influence the antagonistic interaction of these elements in plants, with the presence or higher concentration of one chemical element inhibiting the uptake, transport, or accumulation of another element into plant metabolism. These bonds usually occur through competing mechanisms in root cells, ion transport systems, or chemical interactions in the soil. Other investigations have also determined iron correlation with other elements in plants [52].

Dependencies for the filler dataset (removed elements with missing values) are shown in Figure 2. The following correlations were obtained: 0.51 for chlorine (Cl) and potassium (K), a significant positive correlation; −0.48 for chlorine (Cl) and silicon (Si), a significant negative correlation; −0.45 for chlorine (Cl) and iron (Fe), a significant negative correlation; and 0.39 for chlorine (Cl) and cadmium (Cd), a significant positive correlation.

The significant positive correlation between Cl and K (0.51) shows that as the amount of chlorine in plants increases, the concentration of potassium increases as well. This interaction indicates these elements’ common uptake mechanism or their interaction in plant physiological processes. Potassium influences plant growth and root development, and previous research has confirmed that potassium might increase the accumulation and transport of certain microelements [4,53,54].

A significant negative correlation was indicated for Cl and Si (−0.48). This showed that when chlorine increases in plants, the amount of silicon decreases. Different uptake pathways or functions of these elements in plants might explain this finding. For instance, chlorine may be actively incorporated due to salt accumulation mechanisms, and silicon might be incorporated due to its role in strengthening plant tissues. Other studies have shown that silicon can control the uptake of various elements [55,56].

The significant negative correlation between Cl and Fe (−0.45) indicates that greater amounts of chlorine caused a reduction in iron in plants. This may result from the antagonist interaction mechanism of these elements or different sources of their absorption. For instance, chlorine can be obtained from disinfectants (sodium hypochlorite), while iron is mainly absorbed from mineral compounds. The significant positive relationship between Cl and Cd (0.39) indicates that increasing amounts of chlorine and cadmium in plants might derive from a common source of pollution or similar accumulation mechanisms of these elements. Interactions between cadmium and chlorine may be related to the soil composition or environmental pollution by disinfectants, affecting their availability in plants.

Figure 3 shows the dependencies for aggregated data (mean values) by sample numbers. This figure shows a correlation matrix indicating the relationships between chlorine (Cl) and other microelements (Cd, Si, K, Ca, Fe, Zn, and P) in plants. The trends remained the same as in previous figures: a significant positive relationship for Cl and K (0.55); a significant negative correlation for Cl and Si (−0.54); a significant negative correlation for Cl and Fe (−0.51); and a significant positive correlation for Cl and Cd (0.53). This confirms a previous analysis [57] that showed that with positive correlations, when one element increases, the other element increases. Meanwhile, a negative correlation shows that when one element increases, the other element decreases.

The interactions with chlorine (Cl) detailed in Figure 3 indicate significant positive correlations between chlorine (Cl) and potassium (K) and cadmium (Cd), indicating that a higher amount of chlorine in plants is associated with higher amounts of potassium and cadmium. This might be explained by a common source of contamination or similar transfer mechanisms in plants. Chlorine (Cl) and silicon (Si) showed a negative correlation, which means that when the amount of chlorine increases, the amount of silicon decreases. This might be related to the protective properties of silicon against salt toxicity—silicon can reduce the accumulation of chlorine in cells. The significant negative correlation between chlorine (Cl) and iron (Fe) reveals that plants with more chlorine contain less iron. This might be related to competition for uptake or changes in soil properties affecting the availability of these elements. The correlation between chlorine (Cl) and phosphorus (P) was weak. It can be assumed that chlorine does not significantly affect the distribution of phosphorus in plants. Other investigations [58] have revealed that phosphorus interacts differently with microelements. This can be explained by the fact that the relationship between chlorine and phosphorus (weak correlation) may depend on specific conditions.

An analysis of the interactions between chlorine and other elements in plants revealed positive correlations with potassium (K) and cadmium (Cd), indicating that these elements may accumulate together or be affected by common processes. Negative correlations with silicon (Si) and iron (Fe) indicate possible competition for uptake or antagonistic interactions. Chlorine may be important in ion balance, but its excess may interfere with the absorption of microelements (e.g., Fe).

Correlation analysis assesses the interactions between chlorine and certain microelements, which can affect their uptake by plants. For instance, the presence of one element promotes the uptake of another or inhibits the accumulation process. This might affect the effectiveness of phytoremediation measures and the selection of the plants that best retain contaminants. Studies have shown that chlorine uptake might be accompanied by other elements [54].

### 2.2. Cluster Analysis

**Figure 4:**
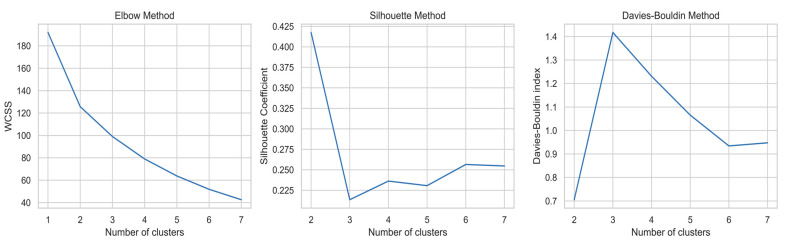
Determination of the optimal number of clusters using three methods: the Elbow method, the Silhouette coefficient, and the Davies–Bouldin index. All three indicate that two or three clusters best represent natural groupings in the dataset.

Cluster analysis was performed to determine the behavior of chlorine and other elements (i.e., similarities, relationships, and mutual influence). Figure 4 presents the results of cluster number evaluation using three different methods. The Elbow method indicates a noticeable decrease in within-cluster variance up to three clusters, after which the improvement becomes minimal. The Silhouette method shows the highest value at two clusters, but the difference between two and three clusters is small, suggesting that both options are acceptable. Similarly, the Davies–Bouldin index reaches its lowest value at two clusters, but the value for three clusters is very close. Considering all methods, three clusters were selected as the most balanced and interpretable option for the present research, reflecting meaningful groupings in the dataset. This allows for the evaluation of elements’ composition in plants, as well as their properties and impacts. This is important for further data interpretation in order to achieve effective phytoremediation. Cluster analysis is often used to assess the behavior of elements, as shown in other studies [59,60].

**Figure 5:**
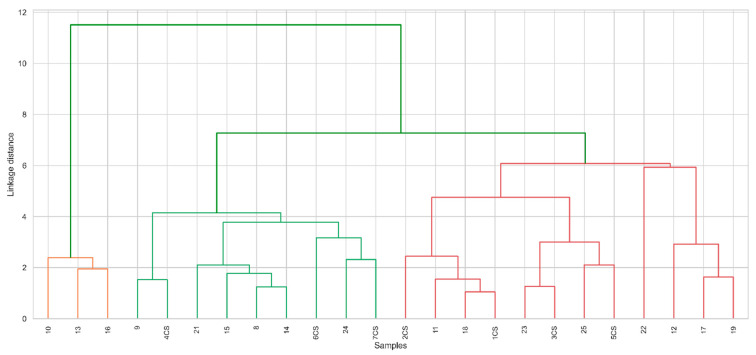
Hierarchical dendrogram displaying the clustering of samples into three distinct groups based on chemical composition. Different colored branches (orange, green, red) represent the three clusters, separated according to the linkage distance threshold used in the clustering. Cluster 0 is high in Cl, K, Ca, and P; cluster 1 is high in Si and Fe; cluster 2 is low in Zn.

Figure 5 shows a dendrogram with hierarchical clustering, which divides the samples into three groups. The average concentrations of microelements in each cluster are given in Table 1. Group 0 (12 samples)—red. This group has a high concentration of chlorine, calcium, potassium, and phosphorus. It also includes silicon, cadmium, and iron. The concentration of zinc in this group is also high. Group 1 (3 samples)—orange. This group is low in chlorine, potassium, and phosphorus but distinguished by a very high amount of silicon and iron. The amount of zinc in this group is also slightly lower. Group 2 (9 samples)—green color. It has an average amount of potassium and calcium but a very low concentration of zinc and silicon, which distinguishes this group from the others. This may indicate that this group has a different chemical or structural complexity. The dendrogram shows that the largest differences between groups are found in chlorine, potassium, and silicon concentrations (Figure 5).

**Figure 6:**
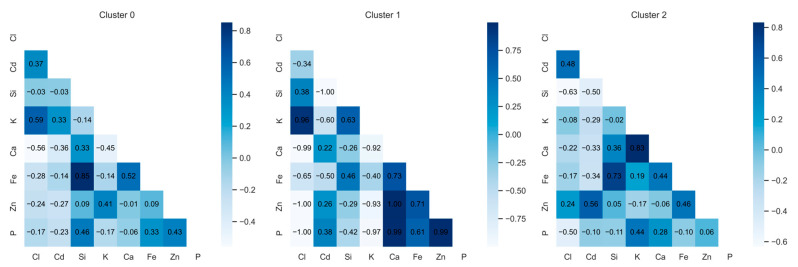
Cluster-specific correlation heatmaps showing the relationships between chlorine and microelements within each group. Each cluster displays unique interaction patterns, indicating the influence of environmental and biological factors.

The dependencies of microelements within the groups are presented in Figure 6. Each cluster has different chemical links, indicating that elements interact differently in different groups. Cluster 1 has the strongest correlations, which may indicate a stronger chemical link between elements. Cluster 2 shows correlations, indicating more associated links between elements. Negative correlations (Si and Cd) may indicate inter-element competition or chemical antagonisms. Cluster 0 shows a positive correlation between chlorine (Cl) and potassium (K) (0.59). It can thus be concluded that chlorine levels are related to plants’ potassium availability. The weak negative correlation between chlorine (Cl) and zinc (Zn) (−0.17) indicates possible antagonism between chlorine and zinc, which may affect plants’ micronutrient uptake. Cluster 1 indicates a negative correlation between chlorine (Cl) and cadmium (Cd) (−0.34), which shows that the concentration of cadmium can decrease with a high amount of chlorine. This can be beneficial because cadmium is toxic to plants, so the presence of chlorine can help reduce damage. A strong negative correlation between chlorine (Cl) and phosphorus (P) (−1.00) may indicate that high levels of chlorine reduce phosphorus’s availability to plants, which may negatively affect root development and photosynthesis. Cluster 2 shows a positive correlation between chlorine (Cl) and cadmium (Cd) (0.48). This reveals that, unlike cluster 1, the ratio of chlorine to cadmium is positive, which may indicate that higher levels of chlorine may promote cadmium accumulation in certain environments. This can have adverse effects on plants, as cadmium is toxic. The weak negative correlation between chlorine (Cl) and iron (Fe) (−0.17) may indicate that higher amounts of chlorine reduce the availability of iron to plants, which can cause iron chlorosis (the yellowing of leaves due to iron deficiency) Other studies [61] have shown high iron uptake in plants, which confirms the metabolism of various microelements. The chlorine correlation shows that chlorine can change the availability of microelements in the soil, and the weak correlation between chlorine and iron can be weakened by other factors (e.g., the phosphorus concentration, etc.). Considering that the mechanisms of iron transport in plants are still not fully studied [62], the weak correlation between Fe and Cl may be related to indirect or complex interactions, which are not reflected in simple concentration relationships. This may be important in future studies analyzing chlorine movements in plants.

Cluster analysis revealed different trends in the distribution of certain elements in plants and their abilities to accumulate or remove pollutants. The samples naturally fall into three groups that differ in terms of chemical composition (Figure 6): Group 0: a high concentration of chlorine, calcium, potassium, and phosphorus; Group 1: a low concentration of chlorine and potassium, but a high content of silicon and iron; Group 2: a medium concentration of potassium and calcium, but a very low content of zinc and silicon. This evaluation helps to identify the effectiveness of phytoremediation.

Previous studies [57] have analyzed the interactions of chlorine with elements in sediments to identify the main correlations. Both analyses revealed the different behaviors of chlorine. The interaction of chlorine with microelements in stormwater sediments depends on the environment. In stormwater sediments, chlorine (Cl) strongly interacts with iron (Fe) and calcium (Ca), forming compounds (FeCl_3_ and CaCl_2_) that can promote surface corrosion. In plants, chlorine showed both positive and negative correlations with microelements, and its excess can promote or limit the uptake of certain elements. Excess chlorine can have adverse effects. In the case of stormwater, the use of chlorine, especially in the form of disinfectants, causes environmental pollution and corrosion. In plants, excessive chlorine reduces the uptake of silicon (Si) and iron (Fe), which can impair plant resistance to adverse conditions. Microelement interactions are very complex and depend on environmental conditions. Stormwater analysis showed a strong correlation between Cl and Fe; in plants, the correlation between Fe and Cl was negative. Cadmium (Cd) accumulation in plants may depend on the residual chlorine content, but this may vary depending on environmental conditions. Residual chlorine can promote the accumulation of certain elements but simultaneously limit the uptake of other important microelements. In the case of stormwater sediments, this is related to pollution and corrosion, and in plants, this is related to nutritional processes and plant resistance. These findings are important for future research on stormwater treatment using green infrastructure.

## 3. Materials and Methods

This study investigated plants’ phytoremediation capacity to retain residual chlorine and analyzed the interactions between residual chlorine and other microelements in plants. In connection, an experimental model of a rain garden was developed. The results were analyzed using the Python programming language in the following order: in the initial stage, a correlation analysis of the data was conducted to assess the interactions between chlorine (Cl) and microelements. Later, cluster analysis was performed to ensure the reliability of the model via additional grouping of data to identify possible group differences and assess the dependencies in separate groups. This provided an opportunity to more accurately assess the possible trends, the connections between chlorine and other microelements, and the peculiarities of their distribution in plants.

### 3.1. Field Experiment

#### 3.1.1. Location Area and Experimental Model Installation

**Figure 7:**
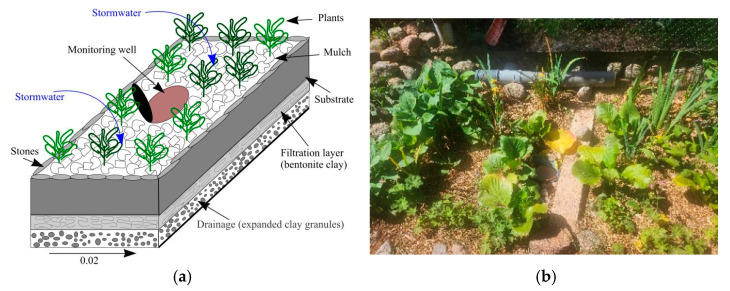
Principle scheme (**a**) and pictures (**b**) of the experimental rain garden.

The research area is located in the Vilnius region, Buivydiskes, Lithuania (approximate geographical coordinates: 54.71552° N, 25.19732° E). A small experimental rain garden (1 × 3 m) was installed close to the stormwater pipe and impervious surface at the end of March 2024. The investigated rain garden consists of the following main layers: a drainage layer (expanded clay granules, 10 cm), a filtration layer (bentonite clay, 20 cm), a soil substrate and plants (7 species), and a mulch layer (pine shavings, 10 cm). All materials used in the experimental rain garden were selected based on previous research [24,46]. In order to prevent landslides, the rain garden slopes were reinforced with stones (Figure 7).

The aim of the experiment was to assess the ability of plants to retain residual chlorine in a rain garden compared to the same plants growing in a control environment—a regular garden with typical garden soil.

#### 3.1.2. Plant Selection and Sampling

Plants for residual chlorine retention were selected based on the recommendations outlined in previous scientific research [42,43,44,45]. Plants were selected considering the following parameters: first, local plants, which can grow in various conditions including dry, wet, sun, or shade. Second, plants that do not require intensive care and have excellent flowering and foliage characteristics. Third, plants that have adsorption properties and are applicable for pollution reduction through phytoremediation [63,64]. Studies recommended using different types of plants in rain gardens that adapt well to the local conditions, differ in height, and form an aesthetically pleasing composition. Rain garden plants act as natural filters that help to retain and reduce environmental pollution, improve water quality, decrease surface runoff, contribute to ecosystem balance and the development of biodiversity, and help to mitigate climate change. The surface of the experimental rain garden was covered with mulch to reduce evaporation and prevent the growth of weeds. A description of the plants is provided in Table 2.

Plants were vegetated from March to the end of October. During the experiment, impermeable surfaces were permanently disinfected using a sodium hypochlorite solution. The concentration was prepared according to WHO recommendations of 1000 ppm [27] to ensure constant application. Changes in plants were observed visually every two weeks by comparing the same plant species growing in an experimental rain garden (i.e., with additional disinfection by sodium hypochlorite) and in regular soil (without specific pollution). Visual changes were monitored and included the following parameters: changes in leaf color (e.g., yellowing or spotting—a possible sign of chlorine or micronutrient imbalance), growth changes, differences in root system development (if visible, e.g., in grounded plants), changes in plant shape or stiffness, and signs of drying or wilting.

At the end of October, plants were harvested, dried, ground, and transported to the laboratory for analysis. The residual chlorine and microelement measurements in plant samples were conducted in the laboratory (Opole University, Opole, Poland) using the Niton XL5 Plus handheld XRF analyzer (Thermo Scientific, Waltham, MA, USA).

### 3.2. Data Analysis

Data analysis was performed using the Python programming language (version 3.9) due to its flexibility, wide range of statistical libraries, and suitability for reproducible scientific computing [65,66]. Following previous research [57], the analysis included data cleaning, correlation analysis, and hierarchical clustering to identify patterns in the interaction between residual chlorine and microelements in plants.

**Figure 8:**
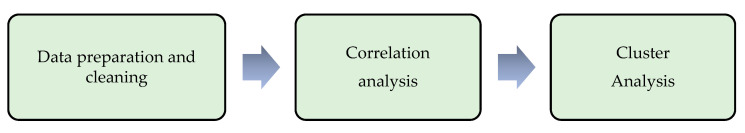
The key steps of the data analysis process.

Data analysis was carried out in the following order (Figure 8):

Data Preparation and Cleaning: the initial dataset included measurements of elemental concentrations (e.g., Cl, Cd, Fe, Si, and K) across different plant samples. Prior to statistical analysis, the data were inspected for missing values. Variables representing specific elemental concentrations and variables with over 25% missing entries were excluded to reduce bias and ensure the accuracy of results. The remaining data were standardized (mean-centered and scaled to unit variance) to account for differences in measurement scales and ensure compatibility across statistical methods.

Correlation Analysis. Correlation matrices were calculated using Pearson’s correlation coefficient (r) to identify linear relationships between chlorine and other microelements. Pearson’s correlation coefficient (r) was calculated in order to investigate relationships between the selected elements and define relationships between the remaining elements. The correlation matrix was computed to quantify linear associations between variables, providing insights into potential dependencies. To enhance interpretability, a heatmap was generated, which visually represents the correlation coefficients. This visualization facilitated the identification of strongly correlated variables that could influence subsequent regression modeling and feature selection. Pearson’s correlation coefficient (r) was calculated in order to investigate relationships between the selected elements.

$$r_{xy}=\frac{n\sum x_{i}y_{i}-\sum x_{i}\sum y_{i}}{\sqrt{n\sum x_{i}^{2}-{\left(x_{i}\right)}^{2}}\sqrt{n\sum y_{i}^{2}-{\left(y_{i}\right)}^{2}}}$$

where n is the number of observations, $x_{i}$ is the value of x (for the *i*-th observation), and $y_{i}$ is the value of y (for the *i*-th observation).

The Pearson coefficient measures the linear dependence between two variables and ranges from −1 to 1 [67], where

- Values close to 1 indicate a strong positive correlation;
- Values near −1 suggest a strong negative correlation;
- Values around 0 imply no linear relationship.

The analysis was conducted in three phases: using the original dataset with missing values ignored, a filtered dataset excluding samples with missing values, and aggregated data based on the mean concentrations by sample group. Statistical significance was tested (α = 0.05) for each coefficient using a two-tailed *t*-test, and *p*-values were calculated [68]. Correlation heatmaps were generated using the Seaborn library (version of the Seaborn library 0.13.2) to visualize trends and identify strong positive or negative relationships among elements.

The Cluster Analysis aimed to identify natural groupings within the dataset, and to explore underlying groupings in the dataset, hierarchical clustering was applied [69]. The optimal number of clusters was determined using three standard validation methods [70,71,72]:

- The Elbow method (evaluating the Within-Cluster Sum of Squares);
- The Silhouette Score;
- And the Davies–Bouldin index.

All analyses suggested that three clusters provided the best balance between cohesion and separation. Cluster analysis was performed using Ward’s method and visualized using dendrograms, enabling the identification of patterns in the co-occurrence of elements and distinguishing plant groups with distinct chemical compositions.

Correlation matrices were recalculated within each cluster to assess how microelement interactions varied across groups. These cluster-specific matrices provided insights into environment-dependent behavior, such as potential antagonism between chlorine and micronutrients like iron or silicon, or synergistic uptake patterns with cadmium or potassium.

## 4. Conclusions

The present research showed that chlorine correlates differently with specific elements. Positive Cl correlations (with Cd and K) potentially reveal a common origin and transportation mechanism. Elevated chlorine levels in plants may influence the accumulation of these elements. Negative correlations (with Fe and Si) indicate competing uptake or translocation pathways, or even toxic effects, where an excess of one element inhibits the uptake of another (e.g., excess chlorine may inhibit the uptake of these elements).

Cluster analysis revealed that microelements in plants form distinct groups with specific chemical profiles. For example, high chlorine, calcium, potassium, and phosphorus concentrations may indicate more intensive nutrient uptake or the effects of excessive fertilizers. The presence of low levels of chlorine and potassium, but high silicon and iron, may indicate a specific stress response or a greater role of silicon in strengthening resistance to environmental stressors.

These insights help in distinguishing different plant groups according to their chemical composition, which may be useful in future research on soil quality and microelement distribution patterns, as well as their uptake and interactions in plant tissues and soil.

Future research can apply these findings to select plant species that effectively retain residual chlorine and other micropollutants which may be present in stormwater.

## Figures and Tables

**Table 1:** Average concentrations of microelements (±standard deviation) in each cluster (ppm).

Cluster	Cl	Cd	Si	K	Ca	Fe	Zn	P
0	18,540.58 ± 11,528.49	16.44 ± 1.61	7692.49 ± 6126.84	43,217.17 ± 21,348.37	65,431.30 ± 19,973.15	1453.98 ± 1266.47	87.64 ± 74.76	2675.41 ± 1519.74
1	492.42 ± 118.81	6.64 ± 2.51	117,910.33 ± 25,123.66	12,797.76 ± 1611.16	12,437.86 ± 6349.21	9779.36 ± 1570.38	78.80 ± 66.60	1023.27 ± 302.28
2	11,232.99 ± 5090.17	16.69 ± 2.55	17,446.51 ± 15,066.89	34,056.66 ± 21,563.29	22,782.48 ± 4489.93	3535.04 ± 3630.28	46.01 ± 35.26	1323.74 ± 796.05

**Table 2:** A description of the plants.

Plant	Amount	Description
*Iris pseudacorus*	9	Perennial plant. It can be planted in wet coastal areas and in shallow water; it grows well both in the sun and in semi-shade.
*Bergenia crass folia*	8	A perennial plant with medicinal properties. The stem is strong and thick, and the leaves are glossy, massive, and remain green in winter. It grows best in fertile soil, not too dry, in shade or partial shade.
*Primula veris*	6	A perennial plant; it is best planted in a sunny place or in partial shade, in fertile, moderately moist soil. Dry, sandy soil is not suitable.
*Geranium macrorrhizum* L.	12	Perennial plant suitable for carpet planting in larger areas. Grows well under old trees in full shade, and it grows well in full sun, but the plants in full sun become smaller and finer.
*Ajuga reptans*	8	Perennial herbaceous carpet plant that grows well in a sunny place.
*Ligularia*	2	Perennial plant that likes fertilizer, moist soil, and semi-shade. It does not require care.
*Hosta sieboldiana*	Planting area: 2 × 110 cm × 14 cm	A perennial flower. It can grow in wet, moderately wet, and dry soils.

## Data Availability

The data presented in this study are available upon request from the corresponding author.
